# A Case of Li-Fraumeni Associated Thymoma

**DOI:** 10.7759/cureus.24602

**Published:** 2022-04-29

**Authors:** Thibacg Sivayoganathan, Sara Kuruvilla, Matthew J Cecchini, Katherina Baranova

**Affiliations:** 1 Medicine, Schulich School of Medicine & Dentistry, London, CAN; 2 Oncology, London Regional Cancer Program, London, CAN; 3 Pathology and Laboratory Medicine, London Regional Cancer Program, London, CAN

**Keywords:** p53 oncogene, thoracic oncology, thoracic pathology, germline tp53 mutation, anterior mediastinal mass, li-fraumeni, thymoma

## Abstract

Thymomas are among the most common cancers of the anterior mediastinum. They rarely occur in patients with Li-Fraumeni syndrome (LFS), a hereditary syndrome that predisposes individuals to cancer and is characterized by mutations in the tumor suppressor encoding gene *TP53*. Here we describe a case of primary thymoma in a woman diagnosed with LFS. We cover the initial presentation and diagnosis, radiological findings, histopathological examination, and management of thymoma. In addition, we review p53 physiology and LFS pathophysiology to explore how *TP53 *expression might differ between the majority of thymomas and in thymomas associated with LFS. This altered pathophysiology may affect management and prognosis due to emerging evidence of increased resistance to conventional treatment, which suggests a need for close monitoring and consideration of novel treatment strategies such as programmed death-ligand 1 (PD-L1) inhibitors.

## Introduction

Li-Fraumeni syndrome (LFS) is a rare genetic cancer syndrome with a primarily autosomal dominant pattern of inheritance caused by loss-of-function mutations of the tumor suppressor gene TP53. LFS can present in multiple individuals of the same family and lead to multiple primary tumors in the same individual. Almost half of the individuals with LFS are diagnosed with cancer by age 30 [1,2]. LFS imparts significant lifetime cancer risk, with one analysis estimating a lifetime cancer risk of 73% for males and 100% for females [3]. Common LFS-associated cancers include breast cancer, sarcomas, brain cancer, adrenocortical carcinoma, and leukemia, but LFS is also associated with an increased risk for other cancers. Thymomas associated with LFS are rare, with one study presenting a single case of thymoma among 286 p53 mutation carriers [4]. We present a case of LFS-associated thymoma with aberrant p53 staining, suggesting a biological mechanism for developing this cancer in the context of LFS.

## Case presentation

A 57-year-old woman presented to the emergency room with swelling in her neck, face, and right arm that persisted for two months before subsequently worsening. The patient was an ex-smoker with a 30-pack-year history and had no former personal history of cancer. However, she had been previously referred for genetic consultation because of significant family history. Specifically, the patient’s sister was diagnosed with LFS-associated breast cancer, and maternal and paternal history was significant for cancer. The genetic consultation revealed a missense mutation of TP53, with a 374C>T nucleotide change and corresponding Thr125Met amino acid change, identical to her sister’s, deemed likely pathogenic confirmed LFS.

**Figure 1:**
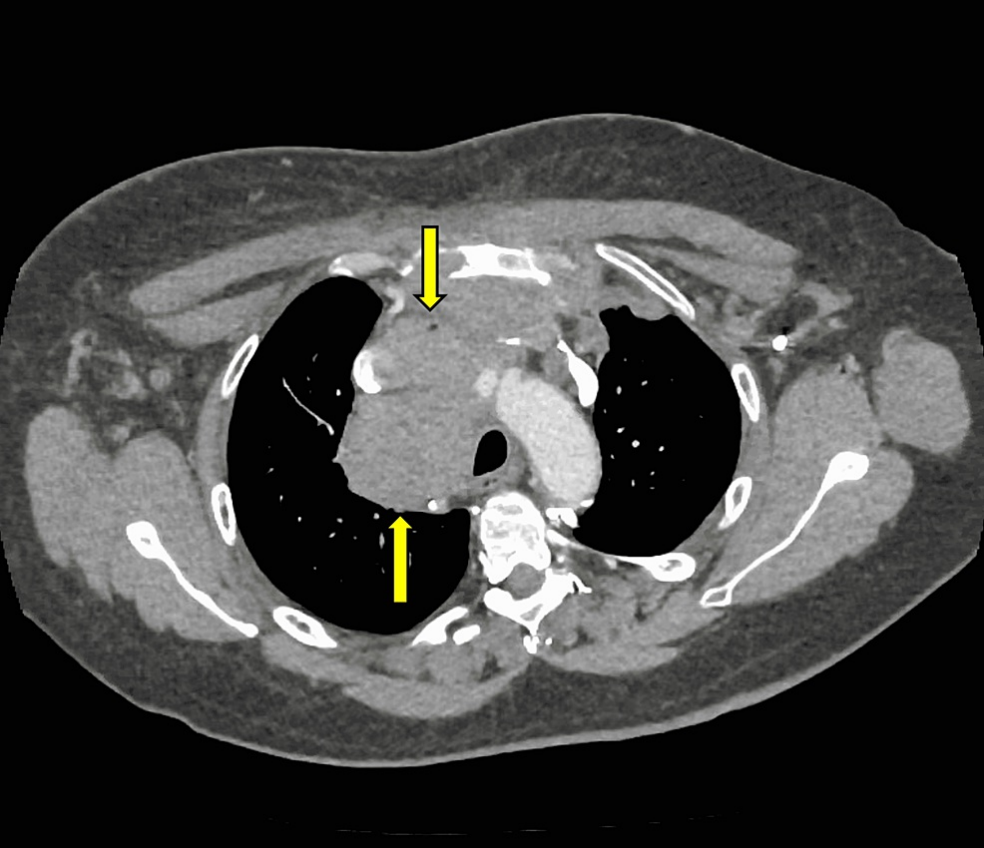
Axial CT thorax image demonstrating an aggressive anterior mediastinal mass. The mass overlaid the sternum and manubrium and was locally invasive, obstructing the superior vena cava and the left brachiocephalic vein. The mass also extended into the left pleural space with likely associated invasion to the left chest wall and left upper lobe.

Upon evaluation, a chest X-ray showed a large superior mediastinal mass. A CT scan confirmed an aggressive anterior mediastinal mass (Figure 1) overlying the sternum and anterior left pleural space. It invaded and obstructed the superior vena cava and left the brachiocephalic vein. Enlarged mediastinal lymph nodes and a small pericardial effusion were evident. There was no evidence of intrabdominal metastatic or primary disease and no brain metastasis.

**Figure 2:**
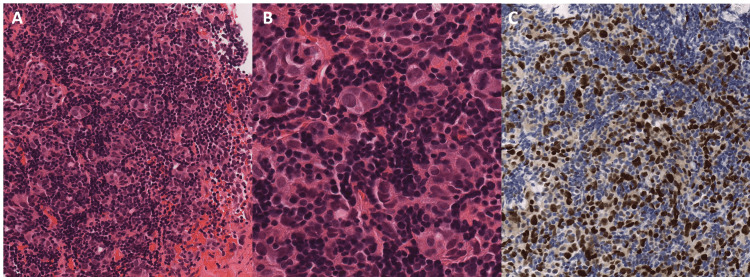
Photomicrographs of tumor (panel A; hematoxylin and eosin; original magnification 10X) showing large atypical cells and admixed thymocytes (panel B; hematoxylin and eosin; original magnification 20X) alongside p53 immunostain, showing strong nuclear positivity in the malignant cells (panel B; immunohistochemistry; original magnification 20X).

Sections from the biopsies were composed of tissue cores with thick fibrous bands and prominent small lymphocytes (Figure 2A). Within the sheets of small lymphocytes, clusters of atypical epithelial cells are present (Figure 2B). The atypical cells were large and highly atypical with hyperchromatic nuclei with fine chromatin. There were also interspersed abnormal cells, and overall, the features were felt to fit within the spectrum of the poorly defined entity of thymoma with anaplasia. The tumor retained some of the organotypic features of the thymus alongside the cytological atypia. There was rare mitotic activity evident. No destructive invasion or desmoplastic stroma was identified. The atypical epithelial cells are positive for pancytokeratin, p63, p53 (strong diffuse, Figure 2B), and cytokeratin AE1/AE3. The small lymphocytes were positive for TdT and CD3. The morphologic features coupled with the immunohistochemical profile supported the diagnosis of a thymoma. Due to the known heterogeneity of thymoma, the subtyping is typically reserved for resection specimens. However, based on tissue sampling in these biopsies, the features are most in keeping with a B2 thymoma. Due to the degree of atypia, the tumor was best classified as a thymoma with anaplasia.

The final diagnosis was locally advanced thymoma (Masaoka stage IVB), resulting in superior vena cava obstruction. The tumor was deemed inoperable. The patient was treated with radiation and six cycles of Cisplatin-Etoposide chemotherapy. Treatment relieved her symptoms and was associated with a reduction in the size of the primary mass, mediastinal lymph nodes, and pericardial effusion. However, the tumor remained unresectable despite treatment due to close association with major cardiac vessels and encroachment into the chest wall. At the eight-month follow-up after her last dose of chemotherapy and radiation, the patient felt well. Her imaging showed a stable appearance of the soft tissue mass in the superior mediastinum. She will have short-term interval imaging to assess for the progression of her disease.

## Discussion

Thymic epithelial tumors are the most common cancer of the anterior mediastinum but are rare compared to other cancers [5,6]. Thymomas are the most common of these thymic epithelial tumors. Diagnosis requires a biopsy, and masses are characterized according to World Health Organization (WHO) classification [7]. Management of thymoma revolves around resection for early-stage tumors, and the prognosis is closely linked to the completeness of the resection. Treatment for thymomas that are locally advanced or metastatic is not curative. Prognosis is also associated with histology, clinical stage, and molecular signature, which may be of significance in the case of thymoma associated with LFS. For its major role in combating genotoxic stress, p53 is also known as the guardian of the genome. Increased levels of p53 result in cell cycle arrest and apoptosis in damaged cells, with the absence of functional p53 leading to the continued survival and DNA damage in cells which can lead to malignant transformation and cancer. Thymic tumors with sporadic TP53 mutations have been suggested to be more aggressive and associated with a worse prognosis in other studies, particularly in thymic carcinomas with associated TP53 overexpression [8-9]. In our case, the pathological diagnosis was thymoma, with the presence of a TP53 germline mutation and overexpression on IHC. This patient has the locally advanced and aggressive disease at presentation, with invasion into multiple surrounding structures and superior vena cava obstruction.

TP53 mutations and associated LFS can also significantly impact management as TP53 mutations have been demonstrated to increase resistance to treatments and increase the likelihood of developing radiation-induced malignancies [10,11]. Evidence suggests that mutant p53 oncoproteins act as homeostatic agents in cancer cells, which may contribute to poor treatment response [12]. Specifically, mutant p53 is stabilized and activated in response to tumor-related stress stimuli. This leads to a positive feedback loop to increase p53 production and initiate adaptive stress responses that promote cancer cell survival and adaptations [12]. In our case, immunohistochemistry demonstrated high levels of p53 staining in the tumor cells, with minimal staining in the background normal thymocytes. p53 signaling pathways are suspected of playing a role in the genesis of sporadic thymoma [13]. Immunohistochemical staining of p53 can be a surrogate marker for TP53 mutations and has been used in ovarian carcinoma [14]. In a study of 10 thymoma patients, increased levels of p53 expression by immunohistochemistry correlated with a worse prognosis [8]. The patient was known to have a germline TP53 mutation; however, the normal thymocytes did not show aberrant staining. This case demonstrated an observational finding of p53 expression in thymoma cells.

There are no specific therapies for LFS-associated thymoma, and treatment is consistent with general guidelines for management, including consideration for surgical resection. However, in this case, the patient was not a suitable candidate for resection due to the extent of the disease. Thus, the patient was treated with radiation and chemotherapy containing Cisplatin-Etoposide to sensitize the tumor to radiation. Considering the potentially worse prognosis of thymomas in the context of LFS and an increased risk of treatment-related malignancy, there may be a need for alternative treatments in this population. This may include consideration of PD-L1 inhibitors, effective in patients with relapsed advanced thymomas [15]. There might also be a future role for tailored treatments targeting the adaptive stress pathways triggered by oncogenic p53 or treatments that restore function to p53 to improve outcomes in these patients [16].

## Conclusions

The presented case highlights that thymoma can be rarely associated with LFS. The pathological findings are of significant interest as the increased p53 levels in the malignant epithelial cells may suggest a worse prognosis and a role in oncogenesis. This may impact the diagnosis, screening, and management of LFS patients with thymoma. Further avenues of research are required to explore this possibility and potential management options tailored for this population.
